# Alterations in circadian rhythms aggravate Acetaminophen-induced liver injury in mice by influencing Acetaminophen metabolization and increasing intestinal permeability

**DOI:** 10.1080/21655979.2022.2079255

**Published:** 2022-05-29

**Authors:** Kun Zhang, Xiude Fan, Xiaoyun Wang, Xiaoge Zhang, Lu Zeng, Na Li, Qunying Han, Yi Lv, Zhengwen Liu

**Affiliations:** a Department of Infectious Diseases, First Affiliated Hospital of Xi’an Jiaotong University, Xi’an, Shaanxi, China; b Department of Hepatobiliary Surgery, First Affiliated Hospital of Xi’an Jiaotong University, Xi’an, Shaanxi, China; c Institute of Advanced Surgical Technology and Engineering, Xi’an Jiaotong University, Xi’an, Shaanxi, China

**Keywords:** Circadian rhythm alteration, oxidative stress, intestinal permeability, Acetaminophen, liver injury

## Abstract

Acetaminophen (APAP) is the most common antipyretic and analgesic drug causing drug-induced liver injury (DILI). Alterations in circadian rhythms can adversely affect liver health, especially metabolic and detoxification functions. However, the effect of circadian rhythm alterations induced by environmental factors on APAP-induced liver injury and the underlying mechanisms are not well known. In this study, a mouse model of circadian rhythm alterations was established by light/dark cycle shift and then treated with excessive APAP. The liver injury indexes, APAP-related metabolic enzymes, and intestinal permeability in mice were evaluated by biochemical analysis, quantitative real-time PCR, enzyme-linked immunosorbent assays, and histopathology. Results showed that circadian rhythm alterations resulted in increased reactive oxygen species (ROS) and malondialdehyde (MDA) and decreased liver superoxide dismutase (SOD), glutathione, and CYP1A2 and CYP3A11 mRNA expression, and increased serum diamine oxidase, lipopolysaccharide, and D-lactate in the mice. Compared with control mice, APAP induced higher serum alanine aminotransferase and aspartate aminotransferase, liver interleukin-1β and tumor necrosis factor-α mRNA, ROS and MDA, lower SOD, glutathione, and UDP-glucuronosyltransferases /sulfotransferases mRNA and more severe liver necrosis and intestinal damage in mice with alterations in circadian rhythms. In conclusion, circadian rhythm alterations by light/dark cycle shift resulted in increased oxidative stress and intestinal permeability in the mice and exacerbated APAP-induced liver injury by influencing APAP metabolization and increasing intestinal permeability.

## Graphical abstract

**Figure:**
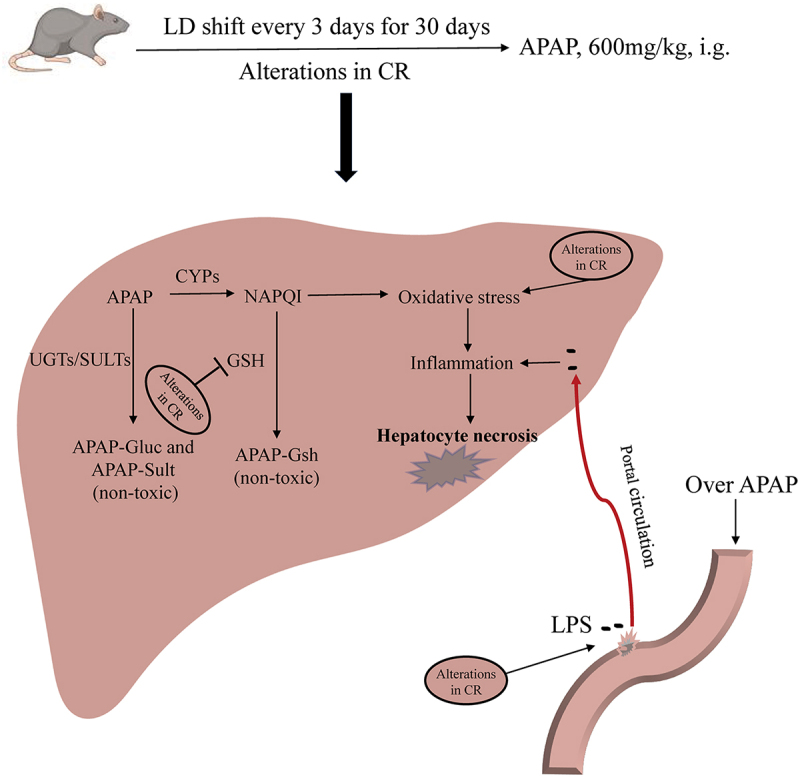

## Highlights

- Circadian rhythm alterations by light/dark (LD) cycle shift caused hepatic oxidative stress and increased intestinal permeability.
- Acetaminophen (APAP) induced more severe liver injury in LD shift mice than in no LD shift mice.
- APAP induced more severe intestinal permeability in LD shift mice than in no LD shift mice.
- APAP induced lower UDP-glucuronosyltransferases/sulfotransferases mRNA in LD shift mice than in no LD shift mice.
- Circadian rhythm alterations exacerbated APAP-induced liver injury via influencing APAP metabolization and intestinal permeability.

## 1. Introduction

Acetaminophen [N-acetyl-p-aminophenol (APAP)], an analgesic-antipyretic agent, is widely used in people of different ages. APAP is considered safe at therapeutic doses, but an overdose of APAP can cause severe liver toxicity and even acute liver failure [1]. APAP hepatotoxicity is the leading cause of acute liver failure in the United States [2], one of the main causes of acute liver failure in industrialized countries [3], and the main reason for drug-induced liver injury in many countries [4]. Therefore, it is meaningful to study the factors affecting APAP-induced liver injury and the underlying mechanisms.

The mammalian circadian rhythms (CR) are hierarchical systems, including the pacemaker clock in the suprachiasmatic nucleus (SCN), the non-SCN brain and peripheral clocks, and cell-autonomous oscillators in almost all cell types [5]. The principal circadian pacemaker in the SCN is regulated by the light/dark (LD) cycles [6]. It transmits signals to cell-autonomous oscillators in tissues, which induce the expression of many genes and regulate various biochemical and physiological rhythms [7]. For example, CR regulates the periodic expression of drug processing genes and transcription factors in the mouse liver [8] and regulates intestinal cell proliferation, colon movement, and nutrient absorption [9–11]. Therefore, alterations in CR may cause adverse health outcomes. It has been reported that alterations in CR may increase the symptoms of irritable bowel syndrome and the risk of colorectal cancer [12,13], lead to oxidative stress and inflammatory responses in the nervous system [14] and promote alcohol-induced steatohepatitis, intestinal leakage, and endotoxemia in mice [15]. Several studies also remind us that CR also plays an essential role in APAP-induced liver injury. APAP-induced acute liver injury manifests diurnal changes, and in most cases, APAP administration at night results in more severe liver injury than in the morning [16–18]. However, it remains unknown whether the alterations in CR may influence the severity of APAP-induced liver injury.

Given the above findings, we hypothesized that alterations in CR may exacerbate APAP-induced liver injury. Thus, we in this study aimed to explore the effects and potential mechanisms of CR alterations on APAP-induced liver injury by establishing a mouse CR alteration model. More precisely, our study included two parts. The first part of the study aimed to determine the effects of CR alterations induced by the 12-hour phase shift of the environmental light/dark (LD) cycle on liver injury, drug-metabolizing enzymes, inflammation and oxidative stress, and intestinal permeability in mice. The second part of the study was to compare the severity of APAP-induced liver injury between the CR shift model and the normal CR model after APAP gavage and explore the potential mechanisms. We hope that this study will provide new information on APAP-induced liver injury and provide clinical implications for the safe use of APAP.

## 2. Materials and methods

### 2.1 Animals

C57BL/6 J mice (7–8 weeks) were purchased from the Laboratory Animal Center of Xi’an Jiaotong University. The mice had access to a standard laboratory diet and tap water. Mice were maintained on a constant 12-hour light:12-hour dark cycle (12:12 LD). All mice received humane care in compliance with the institutional animal care guidelines approved by the Ethics Committee of Xi’an Jiaotong University Health Science Center (No. 2020–429). Ethical approvement can be found in the Supplementary Information. The study was carried out in compliance with the ARRIVE guidelines.

### 2.2 Mice model and tissue collection

**Figure 1.**
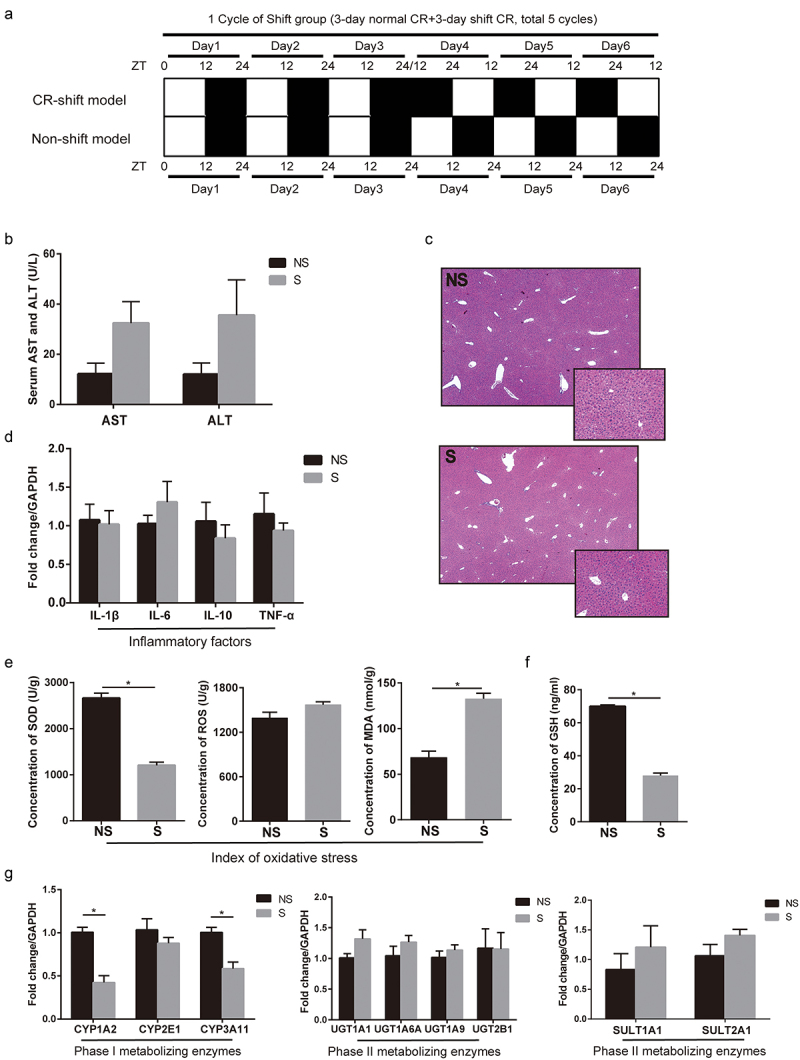
Alteration of the circadian rhythm of the liver in mice by shifted light/dark cycles, decreasing CYPs mRNA expression and increasing oxidative stress. (a) Development of circadian rhythm (CR) shift model by an every 3 days 12:12 hours light/dark phase shift for 30 consecutive days. (b) Serum ALT/AST levels (NS: n = 5, S: n = 5). (c) Representative images (10×, 20×) of liver from H&E. (d) The mRNA levels of IL-1β, IL-6, IL-10 and TNF-α in the liver (NS: n = 5, S: n = 5). (e) Index of oxidative stress: ROS, SOD and MDA (NS: n = 5, S: n = 5). (f) Concentration of GSH in the liver (NS: n = 5, S: n = 5). (g) The mRNA levels of the phase I enzymes (CYPs) and phase II enzymes (UGTs, SULTs) related to APAP metabolism (NS: n = 5, S: n = 5). Results are expressed as the mean ± SEM. Student’s t or Mann-Whitney test was used for statistical evaluation. *P < 0.05. Non-Shifted (NS): Mice without circadian rhythm alterations, Shifted (S): Mice with circadian rhythm alterations.

To study the impact of alterations in CR on the severity of APAP-induced liver injury in mice, we divided the research into two parts. In the first part of this study, 20 C57BL/6 J mice were randomly divided into a CR shift group and a control group. The CR shift model was established by repeating 5 cycles (3 days of normal 12-hour LD phase and 3 days of shifted 12-hour LD phase as one cycle) for 30 consecutive days [15] (Figure 1(a)). In the second part of the study, after the CR shift model was established, at the same time (8:00 am), we gave 600 mg/kg of APAP [19] (Sigma-aldrich, St. Louis, USA, #103-90-2) to the CR shifted mice (n = 19) and control mice (n = 15), respectively, to establish APAP-induced liver injury. Blood was collected at 1 h, 10 h and 24 h after APAP treatment.

Blood was taken from the canthus vein after mice were anaesthetized with Isoflurane (Sigma-aldrich, St. Louis, USA, #26675-46-7) inhalation. The liver and terminal ileum were fixed in 10% neutral formalin for histology, while the other parts were used for biometric measurement. All tissue and serum samples were stored at −80°C until use.

### 2.3 Serum aminotransferase measurements

Mice serum alanine aminotransferase (ALT) and aspartate aminotransferase (AST) were measured at 37°C in the Department of Clinical Laboratory, First Affiliated Hospital of Xi’an Jiaotong University, according to standard methods using a fully automatic biochemical analyzer (HITACHI LABOSPECT 008 AS, Japan).

### 2.4 RNA isolation and quantitative real time-PCR

Total RNA was isolated from mouse liver tissues using TRIzol reagent (Takara BIO INC, China) and used as the template for cDNA synthesis using PrimeScript RT Reagent Kit (Takara, Kusatsu, Shiga, Japan). The ABI StepOneplus Real-Time PCR System (Applied Biosystems) was applied to amplify cDNA with the SYBR Premix Ex TaqTM Kit (Takara, Kusatsu, Shiga, Japan). The relative expression level of each sample’s mRNA was calculated by the 2^−ΔΔCt^ method [20]. GAPDH was used for normalization. The primers used in this study were listed in supplementary materials (Table S1).

### 2.5 Enzyme-linked immunosorbent assays

Quantification of glutathione (GSH), lipopolysaccharide (LPS), diamine oxidase (DAO), and D-Lactate (D-LA) concentration in plasma and reactive oxygen species (ROS), malondialdehyde (MDA), superoxide dismutase (SOD), and GSH in the liver were quantified by enzyme-linked immunosorbent assay (ELISA) using the following kits (all ELISA kits were purchased from Shanghai Jianglai Biotechnology Co., LTD, China): mouse reactive oxygen species (ROS) ELISA Kit (JL20383); mouse MDA ELISA Kit (JL13329); mouse SOD ELISA Kit (JL12237); mouse GSH ELISA Kit (JL20360); mouse LPS ELISA Kit (JL20691); mouse DAO ELISA Kit (JL11855); and mouse D-LA ELISA Kit (JL20161).

### 2.6 Liver and terminal ileum histopathology

Formalin-fixed livers and terminal ileum sections were paraffin-embedded and stained with hematoxylin and eosin (HE). The necrosis degree of hepatocytes was observed under 10× and 20× light microscopy. The degree of intestinal mucosal injury was observed under 20× and 40× light microscopy. At least 5 images were taken per tissue section, and quantification of the hepatic necrosis area was performed using Image-Pro Plus software. Results were expressed as a percentage (%) of necrotic area.

### 2.7 Statistical analysis

In mouse experiments, data were expressed as mean ± SEM. Statistical analysis was conducted using SPSS statistics version 20 (IBM, Armonk, NY). Student’s t or the Mann-Whitney test was used to perform a statistical evaluation of two groups. D’Agostino-Pearson normality test was used to assess normal distribution. *P* < 0.05 was considered statistically significant.

## 3. Results

Since the CR alterations have adverse effects on the metabolic and detoxification functions of the liver, we hypothesized that CR alterations could exacerbate APAP-induced liver injury. To confirm this hypothesis and explore its potential mechanisms, we established a CR alteration mouse model to observe the effects of CR alterations on the hepatic inflammatory response, oxidative stress, drug-metabolizing enzymes, and intestinal permeability in mice. Then, we established APAP-induced liver injury model in the CR alteration mouse model to explore the potential mechanisms by which CR alterations affected APAP-induced liver injury.

### 3.1 Alterations in CR were associated with increased oxidative stress and decreased GSH in mouse liver

CR shift model was developed by every 3 days 12:12 hours LD phase shift for 30 consecutive days (Figure 1(a)). There was no statistical difference in serum AST/ALT levels in the CR shift group compared with the non-shift group (AST, NS: 12.2 ± 4.3 U/L, S: 32.4 ± 8.6 U/L; ALT, NS: 12.0 ± 4.5 U/L, S: 35.6 ± 14.0 U/L) (Figure 1(b)). HE staining showed no obvious inflammation and necrosis in the livers of CR shift mice (Figure 1(c)). Furthermore, CR alterations did not induce the increase of mRNA expression of inflammatory cytokines interleukin (IL)-1β, IL-6, IL-10, and tumor necrosis factor (TNF)-α; (Figure 1(d)). However, the concentration of MDA was higher, and SOD was lower in CR shift mice than those in non-shift mice (Figure 1(e)). The concentrations of GSH in the livers were decreased in CR shift mice compared to non-shift mice (Figure 1(f)). The mRNA expression levels of CYP1A2 and CYP3A11 were lower in mice with CR alterations (Figure 1(g)). The mRNA expression levels of UDP-glucuronosyltransferases (UGT1A6A, UGT1A9, and UGT2B1) and sulfotransferases (SULT1A1 and SULT2A1) had no significant change (Figure 1(g)).

### 3.2 Alterations in CR increased serum DAO, D-LA, and LPS in mice

**Figure 2.**
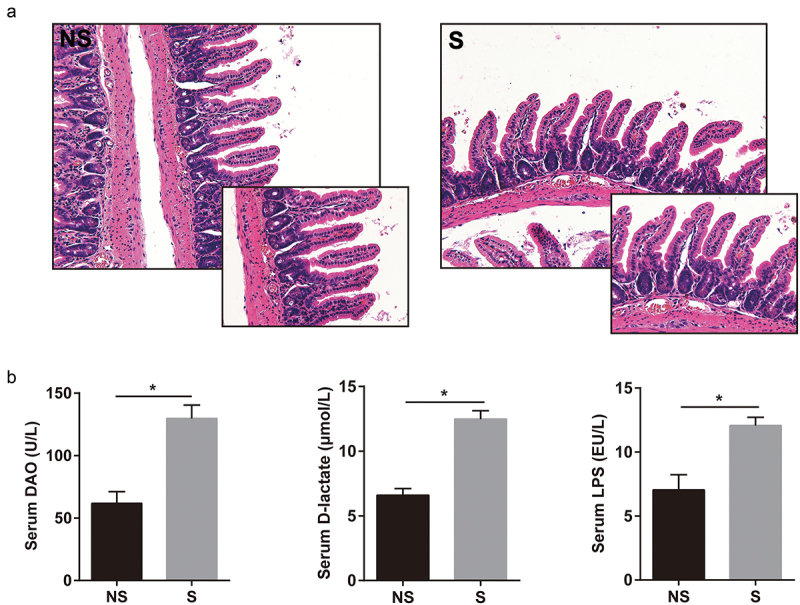
Increased intestinal permeability in mice by circadian rhythm alterations. (a) Representative images (20×, 40×) of terminal ileum from H&E. (b) Serum LPS, D-LA, DAO levels (NS: n = 5, S: n = 7). Results are presented as the mean ± SEM. Student’s t or Mann-Whitney test was used for statistical evaluation. *P < 0.05. Non-Shifted (NS): Mice without circadian rhythm alterations, Shifted (S): Mice with circadian rhythm alterations.

The concentrations of serum DAO, D-LA, and LPS (Figure 2(b)) in the CR shift group were significantly higher than those in the non-shift group (*P* < 0.05). However, no significant change was observed in the histology of terminal ileum sections between these two groups (Figure 2(a)).

### 3.3 Alterations in CR aggravated APAP-induced liver injury in mice

**Figure 3.**
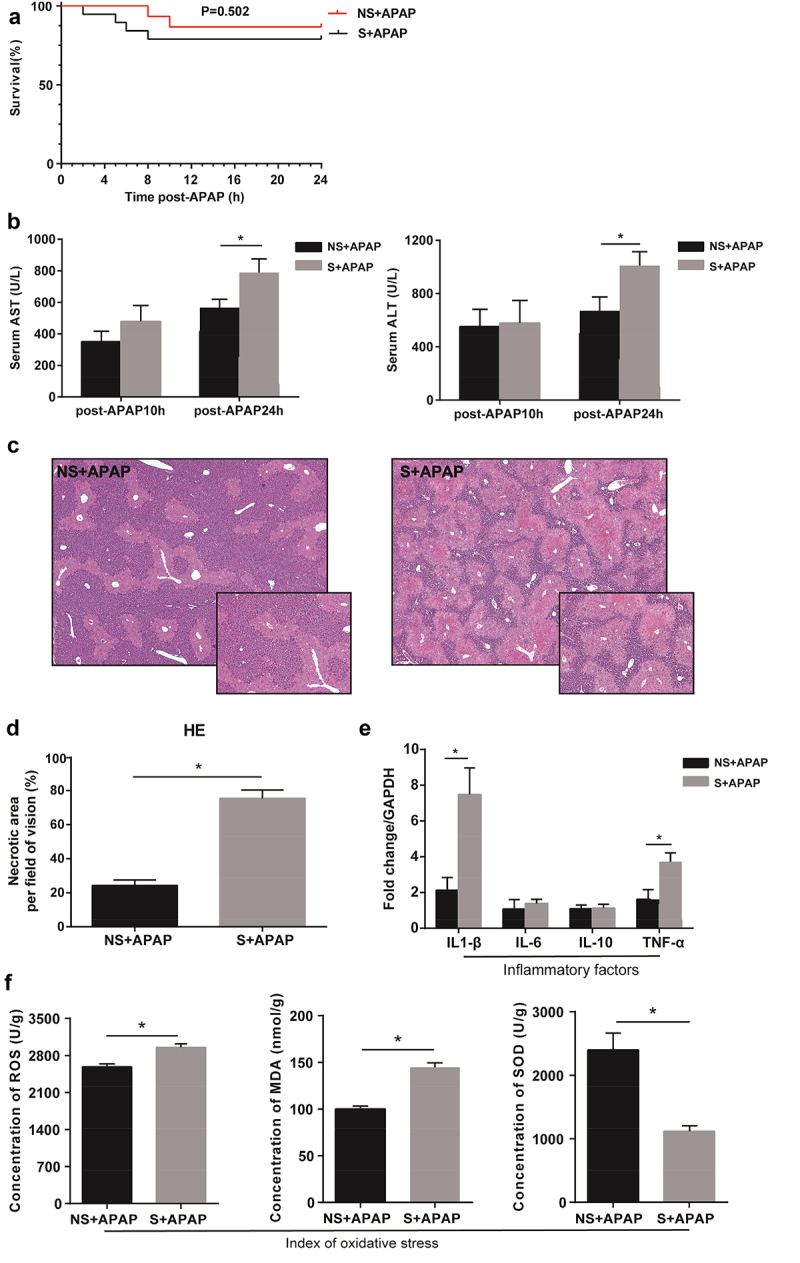
Aggravated APAP-induced liver injury in mice with circadian rhythm alterations. (a) Survival of mice after APAP treated (NS+APAP: n = 15, S+ APAP: n = 19). (b) Serum ALT, AST levels (NS+APAP: n = 7, S+ APAP: n = 5). (c) Representative images (10×, 20×) of liver from H&E. (d) Quantification of the area of hepatocellular necrosis. (e) mRNA levels of IL-1β, IL-6, IL-10, and TNF-α in the liver (NS+APAP: n = 6–7, S+ APAP: n = 5). (f) Index of oxidative stress: ROS, MDA, and SOD (NS: n = 5, S: n = 5).C57BL/6 J mice with or without circadian disruption were treated with APAP (600 mg/kg). Results are presented as the mean ± SEM. Student’s t or Mann-Whitney test was used for statistical evaluation. *P < 0.05. NS+APAP: Mice without circadian rhythm alterations treated by APAP, S+ APAP: Mice with circadian rhythm alterations treated by APAP.

We established an APAP-induced liver injury model using the CR shift and non-shift mice, respectively, to determine the impact of CR alterations on APAP-induced liver injury. After giving 600 mg/kg APAP to CR shift and non-shift mice, 4 mice died in the CR shift group and 2 mice died in the non-shift mice group, and there was no statistical difference in mortality (Figure 3(a)). After 10 hours of APAP administration, AST and ALT levels in the CR shift and non-shift groups increased to 350–550 U/L, but there was no significant difference between the two groups. After 24 hours of APAP administration, the levels of AST and ALT in the CR shift group were significantly higher than those in the non-shift group (*P* < 0.05, Figure 3(b)). APAP-induced liver injury with larger necrotic area was observed in the liver sections of mice in the CR shift group (75.40%) compared to non-shift groups (24.29%, Figure 3(c,d)). In addition, the mRNA expression levels of inflammatory cytokines (IL-1β and TNF-α; Figure 3(e)) and the concentrations of ROS and MDA in the livers were higher and the concentrations of SOD (Figure 3(f)) were lower in CR shift mice than those in non-shift mice.

### 3.4 Alterations in CR could affect the metabolism of APAP

**Figure 4.**
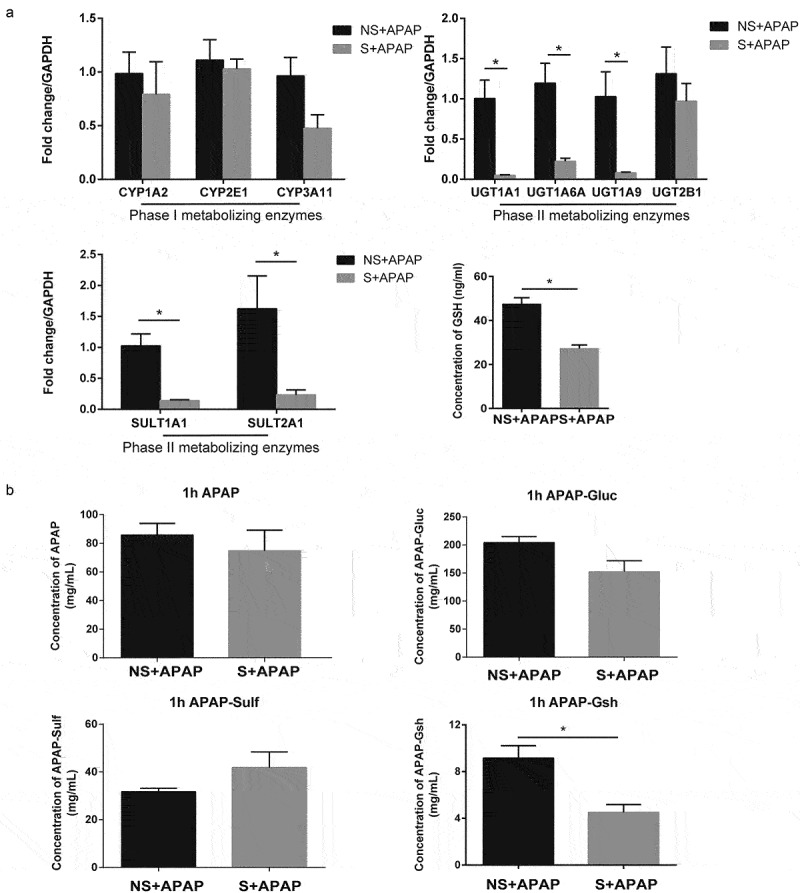
Decreased UGTs and SULTs mRNA expression and significantly increased hepatic oxidative stress in mice with circadian rhythm alterations treated by APAP. (a) The mRNA levels of the phase I enzymes (CYPs) and phase II enzymes (UGTs, SULTs) related to APAP metabolism (NS+APAP: n = 5–7, S+ APAP: n = 5) and concentration of GSH in the liver (NS+APAP: n = 6, S+ APAP: n = 5). (b) Levels of APAP and its conjugates in serum 1 h after mice were treated with APAP (NS+APAP: n = 10, S+ APAP: n = 6). Results are presented as the mean ± SEM. Student’s t or Mann-Whitney test was used for statistical evaluation. *P < 0.05. NS+APAP: Mice without circadian rhythm alterations treated by APAP, S+ APAP: Mice with circadian rhythm alterations treated by APAP.

According to previous studies, the peak blood concentrations of APAP and its related metabolites were reached around 1 hour after APAP administration in both mice and rats [21,22]. When ingested by human adults, APAP is rapidly absorbed from the gastrointestinal tract, and the peak concentration of APAP is reached 30 to 60 minutes after ingestion [23]. Therefore, we measured APAP levels and the metabolites in the serum of mice 1 h after drug administration. The mRNA expression levels of Cytochrome P450 superfamily of enzymes (CYP1A2, CYP2E1, and CYP3A11) were not significantly different between CR shift and non-shift mice (Figure 4(a)). It is worth noting that the mRNA expression levels of glucuronidases (UGT1A1, UGT1A6A, and UGT1A9) and sulfurylases (SULT1A1 and SULT2A1), the concentration of GSH in the liver, and serum APAP-glutathione (APAP-Gsh) concentration were lower in CR shift group than those in the non-shift group (*P* < 0.05; Figure 4(a,b)). However, there were no differences in serum concentrations of APAP, APAP-Gluc, and APAP-Sulf between these two groups (Figure 4(b)).

### 3.5 Alterations in CR induced the worse injury of internal structure of ileum villi and the increased concentrations of serum DAO, D-LA, and LPS

**Figure 5.**
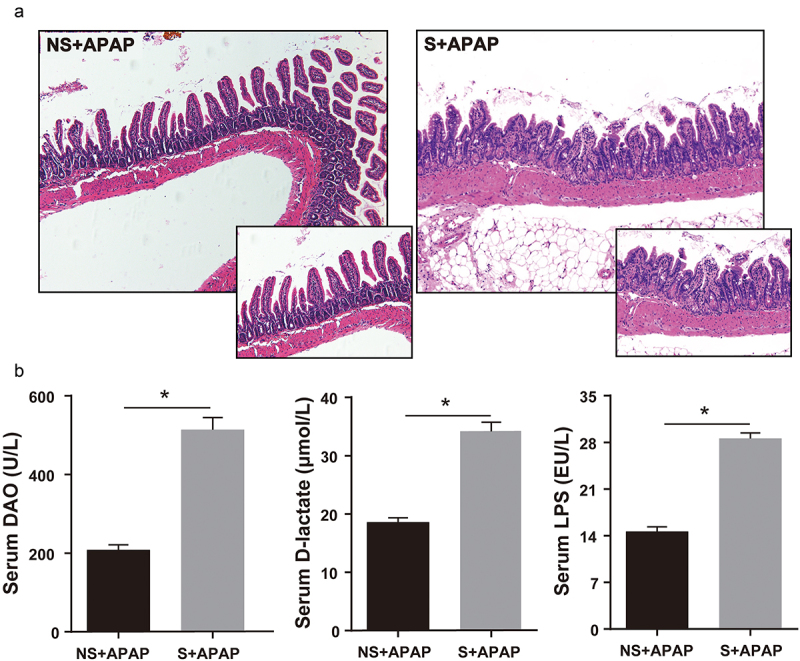
Significantly increased intestinal permeability in circadian rhythm alteration mice treated by APAP. (a) Representative images (20×, 40×) of terminal ileum from H&E. (b) Serum LPS, D-LA, DAO levels (NS+APAP: n = 10, S+ APAP: n = 5). Results are presented as the mean ± SEM. Student’s t or Mann-Whitney test was used for statistical evaluation. *P < 0.05. NS+APAP: Mice without circadian rhythm alterations treated by APAP, S+ APAP: Mice with circadian rhythm alterations treated by APAP.

In the ileum, the injury degree of the internal structure of the ileum villi was worse in the CR shift group than in the non-shift group after gavaging APAP (Figure 5(a)). Furthermore, serum concentrations of DAO, D-LA, and LPS in the CR shift group were significantly higher than those in the non-shift group (*P* < 0.05, Figure 5(b)).

## 4. Discussion

In this study, we first established a mouse model of CR alterations by LD cycle shift to investigate the effects of alterations in CR on liver injury, drug-metabolizing enzymes, inflammation and oxidative stress, and intestinal permeability in mice. The results showed that alterations in CR led to increased hepatic ROS and MDA, decreased hepatic SOD, GSH, CYP1A2 and CYP3A11 mRNA expression, and increased serum DAO, LPS, and D-LA in mice.

Oxidative stress has distinct CR [24]. ROS can cause lipid peroxidation and loss of cell membrane integrity, while MDA is one of the end products of lipid peroxidation [25]. The main antioxidant substances that respond to oxidative stress include SOD and GSH [26]. It has been demonstrated in a study simulating shift work-induced CR disruption in mice demonstrated that CR disruption can lead to mitochondrial dysfunction and increased ROS production in mice [27]. SOD expression and activity were also reduced in the livers of CR gene period2 knockout mice [28]. Our results confirmed the link between alterations in CR and oxidative stress. Interestingly, no severe hepatocyte damage was observed in the livers of the CR alteration mice. It is suggested that the degree of oxidation due to CR alterations does not significantly exceed the clearance of oxidants, which may also depend on the involvement of other antioxidant substances, such as catalase and vitamin C.

The mRNA expression of CYPs, UGTs, and SULTs in the liver also has CR [8]. CYP is a family of enzymes in the liver involved in the metabolism of drugs and other xenobiotics and endogenous compounds. We found that alterations in CR affect the expression of CYP1A2 and CYP3A11 mRNA in mouse liver. However, whether there is a link between the decreased expression of CYP1A2 and CYP3A11 and liver injury needs to be investigated.

The integrity of the intestinal barrier is closely related to CR [29]. DAO is an intracellular enzyme produced by intestinal epithelial cells [29]. Intestinal bacterial fermentation produces D-LA [30]. As components of the cell membrane of Gram-negative bacteria, LPS and DAO and D-LA are indicators of intestinal leakage. A previous study showed that disruption of the circadian clock in mice increases intestinal permeability [15]. This was confirmed by significantly elevated DAO, D-LA, and LPS in the serum of our CR alteration mice.

Then, we compared the APAP-induced liver injury between the CR shift and non-shift mice. Compared with non-shift mice, APAP induced higher AST and ALT, IL-1β mRNA, TNF-α mRNA, ROS and MDA, lower SOD, GSH, UGT mRNA and SULT mRNA, and more severe hepatic necrosis and intestinal damage in CR alteration mice. These results demonstrated that alterations in CR aggravated APAP-induced liver injury in mice. A study found that photoperiod could regulate APAP-induced hepatotoxicity by regulating the expression of the CR gene Per2 [31]. Although the mechanism was different from our study, the role of CR in APAP-induced liver injury was demonstrated in a different way.

The main metabolic processes of APAP include (1) metabolism by UGTs and SULTs to the nontoxic APAP-Gluc and APAP-Sult [32], and (2) metabolism by CYPs to the toxic N-acetyl-p-benzoquinone imine (NAPQI) [33], which can be detoxified by GSH to produce APAP-Gsh. Excess APAP produces excessive NAPQI, which depletes GSH [34,35]. Then, undetoxified NAPQI binds to mitochondrial proteins, leading to irreversible damage caused by downstream oxidative stress [36].

Excessive APAP-induced increases in ROS can lead to JNK activation and increased TNF-α expression through activator protein-1 [37]. Indeed, ROS links multiple environmental exposure factors to disease. In response to environmental exposures, the organism has a continuum from tolerance to ROS through an inflammatory response to cell death and organ damage induced by ROS [38]. At the same time, elevated levels of oxidative stress are accompanied by a significant decrease in SOD and GSH in the corresponding antioxidant system. Reduced GSH in CR alteration mice could affect the conjugation of toxic NAPQI and increase the hepatotoxicity induced by NAPQI. Thus, increased levels of oxidative stress and decreased hepatic GSH caused by alterations in CR may be important pathways that exacerbate APAP-induced liver injury.

After APAP administration, mRNA expression levels of CYP1A2, CYP2E1, and CYP3A11 were not significantly different between CR alteration and normal CR mice. While CYP1A2 and CYP3A11 mRNA expression was decreased in the liver of CR alteration mice before administration. One study confirmed that the expression of CYP3A and CYP1A proteins was significantly elevated in the liver when APAP was administered intraperitoneally to rats at 500 mg/kg [39]. This provides us with a possible explanation that alterations in CR may negatively influence APAP-induced CYP expression. Furthermore, compared to normal CR mice, the mRNA of UGTs and SULTs was not significantly changed in CR alteration mice before APAP administration but was significantly decreased after APAP treatment. This is due to the fact that these UGTs and SULTs are mainly produced by hepatocytes [40], whereas APAP induces more severe hepatic necrosis in CR alteration mice. Additionally, the retinol metabolic process is inhibited in APAP-induced liver injury, which is detrimental to the production of SULTs and UGTs [41,42]. In short, reduced expression of UGTs and SULTs leads to more APAP metabolism by CYPs, resulting in toxic NAPQI and GSH depletion, causing liver damage from downstream oxidative stress.

Intestinal leakage was more severe in CR alteration mice than in non-shift mice before and after APAP administration. Moreover, the intestine of CR alteration mice could be seen to be more significantly damaged under light microscopy after APAP administration. Environmental circadian disruption is a risk factor for susceptibility to liver disease associated with intestinal hyperpermeability and endotoxins [15]. Consistent with previous studies [43,44], excessive APAP causes intestinal damage. Therefore, both CR alterations and excessive APAP were related to intestinal damage. When intestinal permeability increased, bacterial/bacterial products, primarily LPS, are translocated to the liver [45]. In the liver, LPS activates Toll-like receptor 4 signaling in Kupffer cells to produce inflammatory mediators, including TNF-α, IL-1β, and ROS, which cause liver damage [46–48]. This process is known as gut-liver axis injury [49].

Our study has some limitations. Firstly, we established the CR alteration mouse model by LD shift every 3 days for 30 days, which may require longer modeling times for comparison. Secondly, we found that APAP-Gsh was significantly decreased in the CR alteration mice after 1 hour of administration but we did not detect a difference in APAP-Gluc and APAP-Sulf. However, the potentially significant changes of APAP-Gluc and APAP-Sulf in some other time points in the mice over time might be missed because we did not dynamically observe the changes in APAP and its metabolites. Thirdly, although we have studied the mRNA levels of APAP-related metabolic enzymes, the mRNA expression does not directly reflect the enzyme activity. Protein experiments in the changes of drug-metabolizing enzymes should also be performed in future studies. Finally, although we indirectly indicated the accumulation of the APAP toxicant NAPQI through GSH depletion, we did not perform a direct assay for NAPQI. Therefore, more refined studies are needed to explore the molecular mechanisms of CR alterations in increasing the severity of APAP-induced liver injury.

## 5. Conclusions

In conclusion, this study demonstrated that alterations in CR induced by LD cycle resulted in increased oxidative stress in the liver and increased permeability of the intestine in mice, making the mice susceptible to more severe APAP-induced liver injury. In APAP-treated mice with CR alterations, reduced metabolism of APAP by UGTs and SULTs could induce significant oxidative stress, and increased intestinal permeability could lead to bacterial translocation, collectively resulting in more severe liver injury. These findings provide clinical implication that persons with CR alterations might be more sensitive to APAP-induced liver injury although further studies are needed.

## Supplementary Material

Supplemental Material
