# Supplementary material for: Alterations in circadian rhythms aggravate Acetaminophen-induced liver injury in mice by influencing Acetaminophen metabolization and increasing intestinal permeability
Source: Bioengineered. 2022 May 29;13(5):13118–30. doi: 10.1080/21655979.2022.2079255 (PMC9275971; doi:10.1080/21655979.2022.2079255)
Supplement: Supplemental Material [file KBIE_A_2079255_SM8264.doc]

|  | Forward (5'-3') | Reverse (5'-3') |
| --- | --- | --- |
| GAPDH | TTGTCATGGGAGTGAACGAGA | CAGGCAGTTGGTGGTACAGG |
| CYP1A2 | CAAGACCCAGAGCGAGAAGG | GCAGGATGGCTAAGAAGAGGAA |
| CYP2E1 | TGTCATCCCCAAGGGTACAG | GCAGAAACAGTTCCATGCGG |
| CYP3A11 | TTTGGTAAAGTACTTGAGGCAGA | CTGGGTTGTTGAGGGAATC |
| SULT1A1 | CCCTCAGAGTCTGCTGGATCA | TGCTGGTACCACGACCCATAG |
| SULT2A1 | GGGTAACACAAACCTTGTGAAGA | GCTCAAACCATGATCCGAATAGA |
| UGT1A1 | CCCTGCATCTATCTGGCTGA | TGAGACCATGGATCCCAAAG |
| UGT1A6A | GAATATCCCAGGCCGGTCA | CAAAGCCTCAGCAATTTCCA |
| UGT1A9 | GTGGGATCAACTGCCTCCAGA | CTGAGACCATGGATCCCAAAGA |
| UGT2B1 | GACTCCATTGTAAACCAGCCAAAC | GTGGAATCTGGGCAAGAGCA |
| IL-1β | TCCAGGATGAGGACATGAGCAC | GAACGTCACACACCAGCAGGTTA |
| IL-6 | CCACTTCACAAGTCGGAGGCTTA | TGCAAGTGCATCATCGTTGTTC |
| IL-10 | GCCAGAGCCACATGCTCCTA | GATAAGGCTTGGCAACCCAAGTAA |
| TNF-α | ACTCCAGGCGGTGCCTATGT | GTGAGGGTCTGGGCCATAGAA |

**Table S1. Primers used in this study**
